# Cell-Type-Specific Whole-Brain Direct Inputs to the Anterior and Posterior Piriform Cortex

**DOI:** 10.3389/fncir.2020.00004

**Published:** 2020-02-07

**Authors:** Li Wang, Zhijian Zhang, Jiacheng Chen, Anne Manyande, Rafi Haddad, Qing Liu, Fuqiang Xu

**Affiliations:** ^1^Wuhan National Laboratory for Optoelectronics, Huazhong University of Science and Technology, Wuhan, China; ^2^Center for Brain Science, State Key Laboratory of Magnetic Resonance and Atomic and Molecular Physics, Key Laboratory of Magnetic Resonance in Biological Systems, Wuhan Center for Magnetic Resonance, Wuhan Institute of Physics and Mathematics, Innovation Academy for Precision Measurement Science and Technology, Chinese Academy of Sciences, Wuhan, China; ^3^College of Life Sciences, Wuhan University, Wuhan, China; ^4^School of Human and Social Sciences, University of West London, Middlesex, United Kingdom; ^5^The Gonda Multidisciplinary Brain Research Center, Bar-Ilan University, Ramat-Gan, Israel; ^6^University of the Chinese Academy of Sciences, Beijing, China; ^7^Center for Excellence in Brain Science and Intelligence Technology, Chinese Academy of Sciences, Shanghai, China; ^8^Shenzhen Key Lab of Neuropsychiatric Modulation and Collaborative Innovation Center for Brain Science, Guangdong Provincial Key Laboratory of Brain Connectome and Behavior, Brain Cognition and Brain Disease Institute (BCBDI), Shenzhen Institutes of Advanced Technology Chinese Academy of Sciences, Shenzhen-Hong Kong Institute of Brain Science-Shenzhen Fundamental Research Institutions, Shenzhen, China

**Keywords:** anterior piriform cortex, posterior piriform cortex, direct inputs, glutamatergic neurons, GABAergic neurons

## Abstract

The piriform cortex (PC) is a key brain area involved in both processing and coding of olfactory information. It is implicated in various brain disorders, such as epilepsy, Alzheimer’s disease, and autism. The PC consists of the anterior (APC) and posterior (PPC) parts, which are different anatomically and functionally. However, the direct input networks to specific neuronal populations within the APC and PPC remain poorly understood. Here, we mapped the whole-brain direct inputs to the two major neuronal populations, the excitatory glutamatergic principal neurons and inhibitory γ-aminobutyric acid (GABA)-ergic interneurons within the APC and PPC using the rabies virus (RV)-mediated retrograde trans-synaptic tracing system. We found that for both types of neurons, APC and PPC share some similarities in input networks, with dominant inputs originating from the olfactory region (OLF), followed by the cortical subplate (CTXsp), isocortex, cerebral nuclei (CNU), hippocampal formation (HPF) and interbrain (IB), whereas the midbrain (MB) and hindbrain (HB) were rarely labeled. However, APC and PPC also show distinct features in their input distribution patterns. For both types of neurons, the input proportion from the OLF to the APC was higher than that to the PPC; while the PPC received higher proportions of inputs from the HPF and CNU than the APC did. Overall, our results revealed the direct input networks of both excitatory and inhibitory neuronal populations of different PC subareas, providing a structural basis to analyze the diverse PC functions.

## Introduction

The piriform cortex (PC) is located in the ventrolateral region of the forebrain and extends broadly along the anterior to posterior (AP) axis in mammals. As one of the primary olfactory cortex, the PC is involved in encoding odor identification (Gottfried et al., 2006; Howard et al., 2009; Wilson and Sullivan, 2011; Bekkers and Suzuki, 2013; Courtiol and Wilson, 2017), odor associated values or contexts (Gottfried and Dolan, 2003; Calu et al., 2007; Roesch et al., 2007), and odor memory (Zelano et al., 2011; Strauch and Manahan-Vaughan, 2018). Besides, the PC is also implicated in various neurological disorders, such as epilepsy (Loscher and Ebert, 1996; Vismer et al., 2015; Young et al., 2019), Alzheimer’s disease (Samudralwar et al., 1995; Saiz-Sanchez et al., 2015), autism spectrum disorder (Menassa et al., 2017; Koehler et al., 2018) and Parkinson’s disease (Wu et al., 2011).

Previous studies revealed that the PC receives highly converged inputs from distributed glomeruli of the main olfactory bulb (MOB; Vicente and Mainen, 2011), and further synthesizes these odor features into configural odor objects with the help of abundant association fibers within it (Haberly, 2001; Wilson and Sullivan, 2011). Besides olfactory inputs, the PC also receives extensive inputs from the cortical and limbic system (Haberly and Price, 1978; Kowiański et al., 1999; Majak et al., 2004; Illig, 2005). Through these connections, PC can integrate multisensory, emotional and memorial information (Wilson and Sullivan, 2011; Courtiol and Wilson, 2017). In addition, the PC neural activities are also regulated by neuromodulatory axons originating from the cholinergic neurons in the basal forebrain (BF; Wirth et al., 2000; Fletcher and Chen, 2010), the noradrenergic neurons in the locus coeruleus (LC; Bouret and Sara, 2002; Fletcher and Chen, 2010), the serotonergic neurons in the dorsal raphe nucleus (DR; Fletcher and Chen, 2010; Narla et al., 2015), and the dopaminergic neurons in the ventral tegmental area (VTA; Loscher and Ebert, 1996; Shipley and Ennis, 1996). Although the anatomical and physiological evidence revealed some basic connectivity features and information processing mechanism of the PC, the comprehensive neural circuit foundation for functional diversities of the PC remains poorly understood.

The PC is a trilaminar paleocortex that is usually divided into anterior (APC) and posterior (PPC) parts along the AP axis. The borderline is defined by the disappearance of the lateral olfactory tract (LOT) and the thickened layer III in the PPC (Loscher and Ebert, 1996). The APC and PPC play different roles in olfactory processing including odor response and learning (Litaudon et al., 2003; Gottfried et al., 2006; Kadohisa and Wilson, 2006; Calu et al., 2007). For instance, the APC encodes odor identity and anticipation, and can be activated not only by odor stimuli but also by odor associated values or contextual cues (Zinyuk et al., 2001; Gottfried et al., 2006; Kadohisa and Wilson, 2006; Roesch et al., 2007); whereas the PPC seems to encode more associated information for it to be activated in tasks that require encoding of odor similarity or odor quality (Kadohisa and Wilson, 2006; Calu et al., 2007; Howard et al., 2009; Zelano et al., 2011; Bao et al., 2016; Grau-Perales et al., 2019). In addition, accumulating evidence from research has also revealed distinct susceptibilities of different PC subareas to seizure generation (Loscher and Ebert, 1996; Ekstrand et al., 2001; Yang et al., 2006; Vismer et al., 2015). Moreover, the PC comprises glutamatergic principal neurons and γ-aminobutyric acid (GABA)-ergic interneurons. In brief, glutamatergic principal neurons are mainly located in layer II/III in the PC (Suzuki and Bekkers, 2011); GABAergic interneurons, which serve to provide synaptic inhibition of principal neurons and shape stimulus receptive fields, scatter more uniformly across all three layers (Suzuki and Bekkers, 2007, 2012; Luna and Schoppa, 2008; Large et al., 2016). Since the synaptic inhibition of principal neurons are distinct between APC and PPC partly because of GABAergic neurons distribute asymmetrically along the AP range of the PC (Loscher et al., 1998; Luna and Pettit, 2010), revealing the neural connections to specific types of neurons within different PC subareas are essential to shed light on the functional diversities and dysfunctions of the PC.

Previous studies using classical tracers have reported many differences in input connectivity between the APC and PPC (Haberly and Price, 1978; Kowiański et al., 1999). For instance, the APC receives more inputs from the MOB, anterior olfactory nucleus (AON) and orbitofrontal cortex (ORB; Datiche and Cattarelli, 1996; Kowiański et al., 1999; Illig, 2005), whereas the PPC is heavily innervated by the amygdala (AMY; Johnson et al., 2000; Majak et al., 2004). However, traditional tracers are unable to distinguish synaptic connections from pass-by fibers, let alone to exclusively label direct inputs to specific types of neurons.

In the present study, we mapped the direct inputs to glutamatergic principal neurons and GABAergic interneurons within the APC and PPC using the retrograde trans-synaptic tracing system (Wickersham et al., 2007; Wall et al., 2010; Callaway and Luo, 2015). Our results revealed cell-type-specific input patterns to different PC subareas in the whole brain range, and quantitatively compared their input proportions. We found that the input patterns are similar for different PC cell types but diverse for different PC subareas. Our results provide neural connectivity information for further revealing the functional diversities of the PC and its roles in brain diseases.

## Materials and Methods

### Animals

All surgery and experimental procedures were performed in accordance with the guidelines of the Animal Care and Use Committees at the Wuhan Institute of Physics and Mathematics, Chinese Academy of Sciences, and all efforts were made to minimize the number and suffering in experimental animals. Both Vglut2-cre and Gad2-cre mice (Jackson #028863 and Jackson #028867 respectively, gifts from Prof. Liping Wang) were mated with C57BL/6 mice, which were purchased from Hunan SJA Laboratory Animal Company. All animals were housed under standard conditions of humidity and temperature with a 12/12 h light/dark cycle, and food and water were available *ad libitum*. Adult transgenic mice (2–4 months) of both sexes were used for the experiments in the present study.

### Virus Injections

The adeno-associated virus (AAV)-rabies virus (RV) based retrograde trans-synaptic tracers used in this study were generated by BrainVTA (BrainVTA Co., Limited, Wuhan, China), and were stored at −80°C until use. The Cre-dependent AAV helper viruses, composed of AAV-EF1a-Dio-GFP-TVA and AAV-EF1a-Dio-RVG, were packaged into 2/9 serotypes with final titers at about 1.25 × 10^12^ genomic copies per milliliter. The RV-EnvA-ΔG- dsRed was tittered at 3.00 × 10^8^ infecting units per milliliter.

The procedure for virus injection was similar to the one used before in biosafety level 2 animal facilities (Zhang et al., 2017). Briefly, the Vglut2-cre or Gad2-cre mice were anesthetized with sodium pentobarbital (80 mg/kg, i.p.) and mounted to a stereotaxic holder (Item: 68030, RWD, Shenzhen, China) for stereotaxic injection of 80 nl AAV-helper viruses into the APC (coordinates:1.50 mm from Bregma, 2.60 mm lateral from the midline, −4.75 mm from the Bregma surface) or the PPC (coordinates: −1.00 mm from Bregma, 3.60 mm lateral from the midline, −5.25 mm from the Bregma surface). After 3 weeks, 150 nl RV- EnvA-ΔG-dsRed was microinjected into the same site. The mice were kept for 6 days, and then perfused for brain slice collection. Sample size: APC^Vglut2+^, *n* = 6 mice; PPC^Vglut2+^, *n* = 6 mice; APC^Gad2+^, *n* = 4 mice; PPC^Gad2+^, *n* = 4 mice.

### Slice Preparation and Imaging

The mice were overdosed with sodium pentobarbital (100 mg/kg, i.p.), and perfused transcardially with 0.1 M phosphate-buffered saline (PBS, PH 7.4, Sinopharm) followed by PBS containing 4% paraformaldehyde (PFA, Sigma). The brain tissues were carefully extracted from the skull for post-fixation and cryoprotection, then cut into 40 μm coronal sections using the cryostat microtome (Thermo Fisher Scientific) and stored at −20°C.

For input pattern analysis, every sixth section of the brain slices was selected and stained with DAPI (1:4,000, Beyotime), then mounted with 75% glycerol (Sinopharm) in PBS and sealed with nail polish. The brain slices were imaged with the Olympus VS120 virtual microscopy slide scanning system (Olympus).

### Cell Counting and Data Analysis

In this study, the divisions of brain regions and areas were mainly based on the Allen Brain Atlas. In general, the whole brain was divided into eight brain regions, including the isocortex, OLF, HPF, cortical subplate (CTXsp), cerebral nuclei [CNU, consisted of the striatum (STR) and pallidum (PAL)], interbrain [IB, consisted of the thalamus (TH) and hypothalamus (HY)], midbrain (MB) and hindbrain (HB). Each brain region was further divided into several brain areas, even subareas. Supplementary Table S1 shows a detailed list of all related abbreviations.

For cell counting, the number of the starter cells (co-expressing TVA-GFP and EnvA-dsRed) and RV-labeled input neurons (input neurons, only expressing EnvA-dsRed) within each brain area or subarea were quantified respectively in every sixth whole-brain slices by the cell counter plugin in ImageJ. To get rid of the potential leakage of TVA near the injection site, the RV-labeled neurons within the injected PC subarea (ipsilateral APC or PPC) were not counted, but the number of the RV-labeled neurons within another PC subarea in the ipsilateral hemisphere (representing in PC* to avoid confusion) were still quantified. Then, the number of the input neurons within the whole brain or a certain brain region was quantified by adding up the numbers of the input neurons within all related brain areas, with the injected PC subarea excluded.

For quantitative comparison of the distribution patterns of the input neurons across different tracing groups, the normalization was performed relative to the total number of the input neurons in the whole brain/a certain brain region/a certain brain area, and the proportions of whole-brain inputs/a certain brain region inputs/a certain brain area inputs were quantified and analyzed respectively.

For statistical analyses, two-tailed unpaired Student’s *t*-tests and one-way ANOVA tests followed by Bonferroni tests were performed to determine statistical differences using SPSS (version 13.0), with the significance set at *P < 0.05, **P < 0.01 and ***P < 0.001. All data values were presented as mean ± SEM. The related statistics were listed in Supplementary Table S2.

## Results

### Direct Inputs to Glutamatergic and GABAergic Neurons in Different PC Subareas

**Figure 1:**
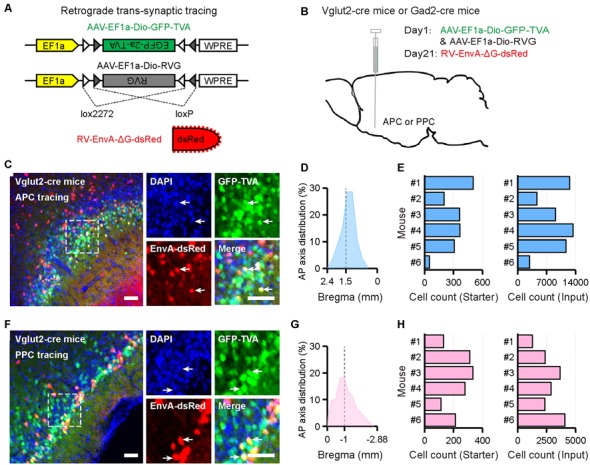
Experimental procedures for the cell-type-specific retrograde trans-synaptic tracing of different piriform cortex (PC) subareas. **(A)** Recombinant adeno-associated virus (AAV) strains and rabies virus (RV). **(B)** Experimental design. **(C,F)** Representative images of coronal brain sections containing the injection sites and the magnifications of the starter cells (**C**, the APC^Vglut2+^ tracing group; **F**, the PPC^Vglut2+^ tracing group). The starter cells were indicated by co-expressing TVA-GFP and EnvA-dsRed. Scale bar: 50 μm. **(D,G)** Distribution patterns of the starter cells in the injected PC subareas as detected along the AP axis (**D**, the APC^Vglut2+^ tracing group; **G**, the PPC^Vglut2+^ tracing group). Gray dashed line, indication of the injection site. **(E,H)** Numbers of the starter cells and input neurons in each mouse. Quantified with every sixth slices (**E**, the APC^Vglut2+^ tracing group; **H**, the PPC^Vglut2+^ tracing group). Starter, starter cells; Input, RV-labeled input neurons.

To identify input patterns of glutamatergic and GABAergic neurons in the APC and PPC, Vglut2-cre mice and Gad2-cre mice were utilized to genetically target distinct neuronal populations, and AAV-RV based retrograde trans-synaptic system was utilized to map the direct inputs to each type of neurons (Figures 1A,B). For both tracing groups, the starter cells were observed near the targeted injection sites (Figures 1C,F). The majority of them were restricted to the injected PC subarea, and distributed widely across the AP range of the injected PC subarea with peak distribution around the targeted injection site (Figures 1D,G). In addition, we found 1,279–13,374 input neurons in each brain (Figures 1E,H). To examine the specificity of the tracing study, the same viruses were injected into the APC of wild-type mice (C57BL/6 mice). We found that, despite a very limited number of the EnvA-dsRed positive neurons near the injection site, no RV-labeled input neuron outside the APC was detected (Supplementary Figure S1). These data suggested a high specificity of the Cre-dependent trans-synaptic property of our viral tracing approach.

**Figure 2:**
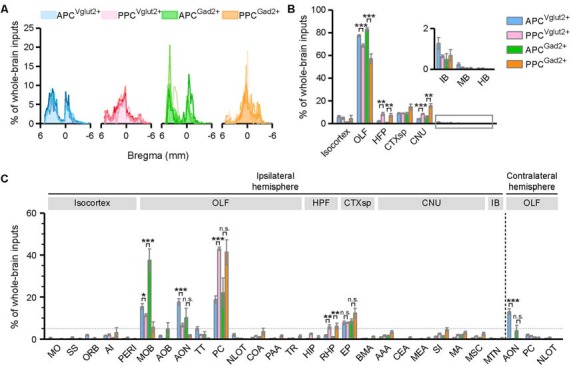
Input patterns to the excitatory glutamatergic and inhibitory GABAergic neurons of different PC subareas. **(A)** Whole-brain distribution of all input neurons along the AP axis. Colored lines, input distribution for the individual mouse; colored line with the shaded area under it, average input distribution. **(B)** Whole-brain distribution of the input neurons within eight brain regions from bilateral hemispheres. **(C)** Whole-brain distribution of the input neurons within 28 brain areas in the ipsilateral or contralateral hemisphere. The brain areas with averaged input proportions greater than 0.5% of whole-brain inputs for either the four tracing groups were selected and illustrated here. ns., no significant difference; *P < 0.05, **P < 0.01 and ***P < 0.001.

When we quantified the whole-brain connections to the APC and PPC, the results showed that excitatory and inhibitory neurons in both PC subareas received extensive inputs from the brain along the AP axis (Figure 2A). To compare the input weight of each brain region across different tracing groups, the number of the input neurons within each brain region from bilateral hemispheres was normalized relative to the total number of the input neurons in the whole brain. For all tracing groups, majority of whole-brain inputs arose from the OLF, followed by the CTXsp, isocortex, CNU, HPF, and IB, whereas the MB and HB were rarely labeled (Figure 2B). It was obvious that, for both types of neurons, the APC and PPC showed distinct features in their input distribution patterns. For instance, the APC received a higher proportion of whole-brain inputs from the OLF, but lower proportions of whole-brain inputs from the HPF and CNU than the PPC did (Figure 2B). To further compare the detailed input features among the four tracing groups, the number of the input neurons within each brain area in the ipsilateral or contralateral hemisphere was normalized relative to the total number of the input neurons in the whole brain. A total of 28 brain areas with averaged input proportions greater than 0.5% of whole-brain inputs for either the four tracing groups were selected and illustrated in Figure 2C. We found that, for both two cell types, the ipsilateral MOB, PC*, AON, EP, and the contralateral AON were the top five input sources and contributed over 72% of whole-brain inputs to the APC in total; while the top five inputs to the PPC came from the ipsilateral MOB, PC*, EP, AON and RHP, and over 67% of whole-brain inputs to the PPC arose from these areas in total (Figure 2C). The APC and PPC showed distinct features in their input distribution patterns in not only ipsilateral but also contralateral hemisphere. For instance, in the ipsilateral hemisphere, the APC received higher proportions of whole-brain inputs from the MOB and AON, but lower proportions of whole-brain inputs from the PC* and RHP than the PPC did (Figure 2C). While in the contralateral hemisphere, the APC received a higher proportion of whole-brain inputs from the contralateral AON than the PPC did (Figure 2C). Although no brain area within the MB and HB was presented and analyzed in Figure 2C for their low proportions of whole-brain inputs, notably, the input neurons in the MB and HB were observed in several key brain areas containing neuromodulatory neurons, including the VTA, dorsal raphe nucleus (DR), and the LC (data not shown). Our results suggested that the input patterns are similar for different PC cell types, but they are diverse in not only ipsilateral but also contralateral hemisphere for different PC subareas. Thus next, we principally focus on the detailed analysis on inputs of the specific PC subareas using Vglut2-cre mice.

### Innervation From the Bilateral OLF to the PC

**Figure 3:**
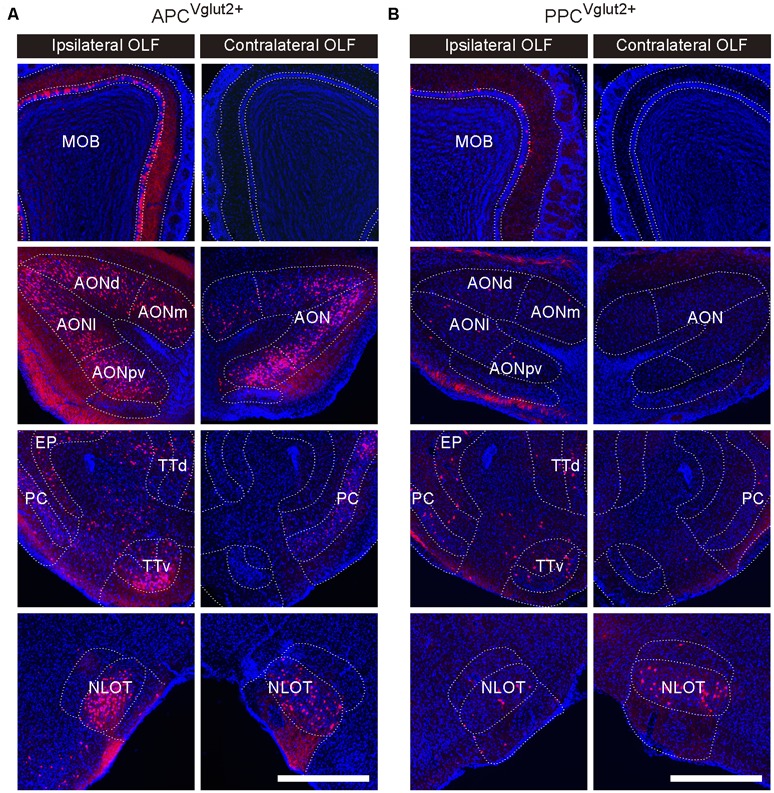
Example images showing inputs from the bilateral OLF to the Vglut2+ neurons of different PC subareas. **(A,B)** In the ipsilateral OLF, the input neurons were found located in the main olfactory bulb (MOB), anterior olfactory nucleus (AON), PC, taenia tecta (TT) and nucleus of the lateral olfactory tract (NLOT), etc. In the contralateral OLF, the input neurons were found located in the AON, PC, and NLOT (**A**, APC^Vglut2+^ tracing group; **B**, PPC^Vglut2+^ tracing group). Scale bar: 500 μm.

**Figure 4:**
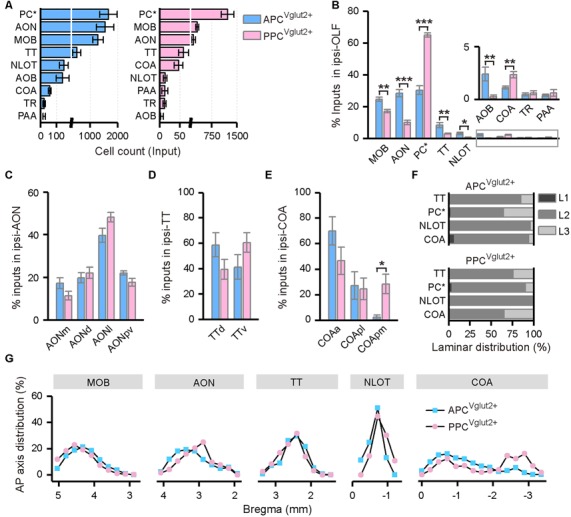
Distribution patterns of ipsilateral OLF inputs. **(A)** Numbers of the input neurons in different areas of the ipsilateral OLF. **(B)** Distribution of the input neurons within the ipsilateral OLF. **(C)** Distribution of the input neurons within the ipsilateral AON. The AON was mainly divided into the dorsal (AONd), medial (AONm), lateral (AONl) and posteroventral (AONpv) parts. **(D)** Distribution of the input neurons within the ipsilateral TT. The TT was divided into the dorsal (TTd) and ventral (TTv) parts. **(E)** Distribution of the input neurons within the ipsilateral cortical amygdalar area (COA). The COA was divided into the anterior (COAa), posterolateral (COApl) and posteromedial (COApm) parts. **(F)** Laminar distribution of the input neurons in the ipsilateral TT, PC, NLOT and COA. **(G)** Distribution patterns of the input neurons along the AP axis in different areas of the ipsilateral OLF. *P < 0.05, **P < 0.01 and ***P < 0.001.

The OLF contributed bilateral innervation to both APC and PPC, but the input neurons distributed more densely in the ipsilateral OLF (Figure 3), including the MOB, accessory olfactory bulb (AOB), AON, PC*, taenia tecta (TT), nucleus of the lateral olfactory tract (NLOT) and cortical amygdalar area (COA), etc. (Figures 3, 4A). Among these brain areas in ipsilateral OLF, the PC*, AON and MOB were the top three input sources to both APC and PPC, and they contributed about 84% and 92% of ipsilateral OLF inputs to the APC and PPC in total respectively (Figure 4A). Our results showed that the AON, MOB, TT and AOB contributed higher proportions of ipsilateral OLF inputs to the APC than to the PPC (Figure 4B). By contrast, the PPC received higher proportions of ipsilateral OLF inputs from the PC* and COA than the APC did (Figure 4B). In most brain areas within the ipsilateral OLF, such as the MOB, NLOT, AON and TT, the distribution patterns of the input neurons were similar between the APC and PPC tracing groups (Figures 4C,D,F,G). While they were distinct within the COA, the posteromedial part of the COA (COApm) contributed a higher proportion of ipsilateral COA inputs to the PPC than to the APC, suggesting a spatial separation of COA inputs to different PC subareas (Figures 4E,G). In addition, the laminar distribution of the input neurons were diverse for the PC*. The APC was innervated by the PC* (refer to ipsilateral PPC here) neurons mainly arising from both layer II and layer III (layer II, 62.15%; layer III, 35.44%); by contrast, the PPC was innervated by the ipsilateral PC* (refer to ipsilateral APC here) neurons mainly arising from layer II (layer II, 86.26%; Figure 4F).

**Figure 5:**
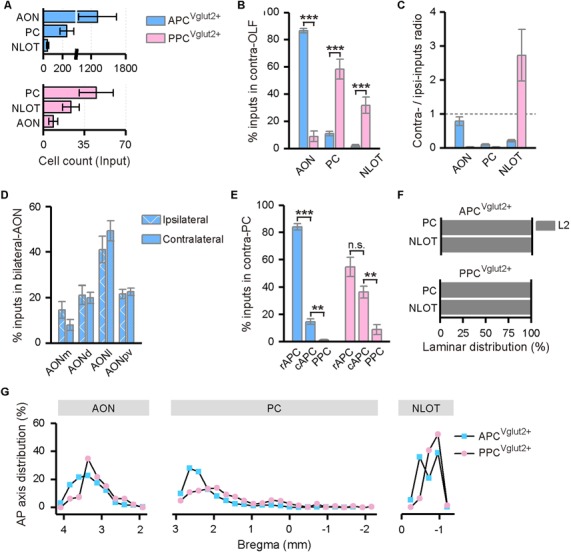
Distribution patterns of contralateral OLF inputs. **(A)** Numbers of the input neurons in different areas of the contralateral OLF. **(B)** Distribution of the input neurons within the contralateral OLF. **(C)** Contralateral-ipsilateral ratio of the input neurons in the AON, PC and NLOT. **(D)** Distribution of the input neurons within the bilateral AON in the APC^Vglut2+^ tracing group. The AON was mainly divided into the AONd, AONm, AONl and AONpv. **(E)** Distribution of the input neurons within the contralateral PC. The APC was further divided into the rostral (rAPC) and caudal (cAPC) parts. **(F)** Laminar distribution of the input neurons in the contralateral PC and NLOT. **(G)** Distribution patterns of the input neurons along the AP axis in different areas of the contralateral OLF. ns., no significant difference; **P < 0.01 and ***P < 0.001.

Contralateral OLF contributed dominant commissural inputs to both APC and PPC (Figure 2C). In the contralateral OLF, the input neurons were distributed specifically in the AON, PC, and NLOT (Figures 3, 5A). Significantly, the APC received much heavier contralateral OLF inputs, with dominant inputs arose from the contralateral AON, than the PPC did (Figures 5A,B). Both input strength and distribution pattern of the input neurons within the contralateral AON were similar to that within the ipsilateral AON in the APC tracing group (Figures 5C,D). In contrast, both the APC and PPC received fewer inputs from the contralateral PC and NLOT (Figure 5A), although the contralateral PC and NLOT acted as the major input sources from contralateral OLF to the PPC (Figure 5B). The input neurons mainly arose from the layer II of the contralateral PC and NLOT (Figure 5F), with obvious ipsilateral innervation preference in most cases, except that the PPC seemed to receive a higher proportion of contralateral NLOT inputs than ipsilateral NLOT inputs (Figure 5C). In addition, for both APC and PPC, the input neurons within the contralateral PC showed predominantly rostral distribution along the AP axis (Figures 5E,G). The APC was mainly innervated by the contralateral APC, especially the rostral part of the APC (rAPC); by contrast, the PPC received commissural inputs from the whole contralateral PC, although the contralateral PPC inputs was much weaker than the contralateral APC inputs (Figure 5E).

### Innervation From the Ipsilateral Isocortex to the PC

**Figure 6:**
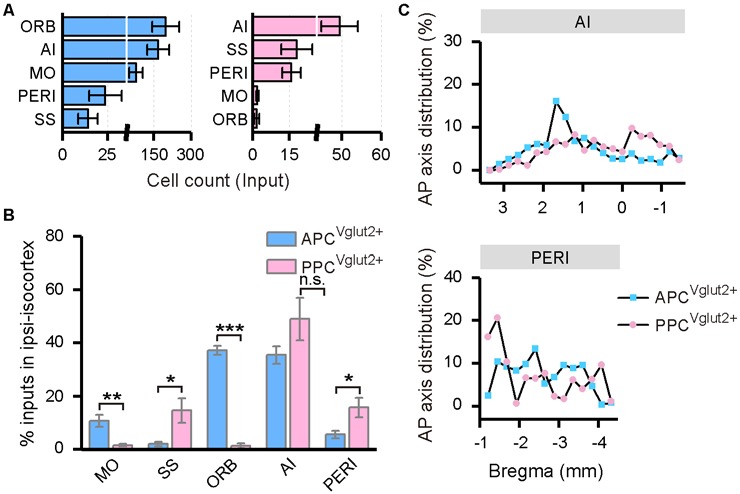
Distribution patterns of ipsilateral isocortex inputs. **(A)** Numbers of the input neurons in different areas of the ipsilateral isocortex. **(B)** Distribution of the input neurons within the ipsilateral isocortex. **(C)** Distribution patterns of the input neurons along the AP axis in different areas of the isocortex. ns., no significant difference; *P < 0.05, **P < 0.01 and ***P < 0.001.

In both the APC and PPC tracing groups, the input neurons were found distributed widely across the ipsilateral isocortex, although a few input neurons were found located in the contralateral side. Thus, only the inputs from the ipsilateral isocortex were analyzed. In the ipsilateral isocortex, the input neurons were mainly observed in the ORB, agranular insular area (AI), somatomotor area (MO), perirhinal area (PERI), somatosensory areas (SS), etc. (Figure 6A). Among these brain areas, the ORB, AI, and MO were the top three input sources to the APC, and about 84% of ipsilateral isocortex inputs to the APC arose from these areas in total; while to the PPC, the AI, SS, and PERI were the main input sources and contributing about 80% of ipsilateral isocortex inputs in total (Figures 6A,B). The subarea distribution patterns of the input neurons within the ipsilateral isocortex were distinct between the APC and PPC tracing groups. The APC received higher proportions of ipsilateral isocortex inputs from the ORB and MO, but lower proportions of ipsilateral isocortex inputs from the PERI and SS than the PPC did (Figure 6B). As the ORB, MO and SS were rarely labeled in either the APC or PPC tracing group (Figure 6A), only the AP axis distribution of the ipsilateral AI and PERI were compared between the two tracing groups. The results showed that, in the AI and PERI, the AP axis distributions of the input neurons were similar between the two tracing groups (Figure 6C).

### Innervation From the Ipsilateral HPF to the PC

**Figure 7:**
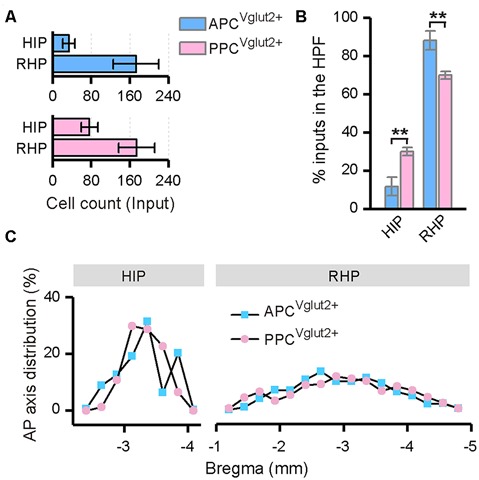
Distribution patterns of ipsilateral HPF inputs. **(A)** Numbers of the input neurons in different areas of the ipsilateral HPF. **(B)** Distribution of the input neurons within the HPF. **(C)** Distribution patterns of the input neurons along the AP axis in different areas of the ipsilateral HPF. **P < 0.01.

Both the APC and PPC received inputs from the ipsilateral HPF, including the HIP and RHP (Figure 7A). In the HIP, the input neurons were specifically located in the ventral part; while in the RHP, the majority of the input neurons were found in the lateral part of the entorhinal cortex (LEC). In both the HIP and RHP, the AP axis distributions of the input neurons were similar between the two tracing groups (Figure 7C). But the subarea distribution patterns of the input neurons within the HFP were distinct. The APC received a higher proportion of ipsilateral HPF inputs from the RHP, but a lower proportion of ipsilateral HPF inputs from the HIP than the PPC did (Figure 7B).

### Innervation From the Ipsilateral PAL to the PC

**Figure 8:**
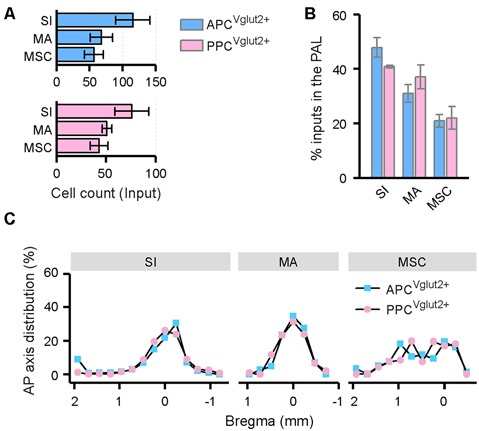
Distribution patterns of ipsilateral PAL inputs. **(A)** Numbers of the input neurons in different areas of the ipsilateral PAL. **(B)** Distribution of the input neurons within the PAL. **(C)** Distribution patterns of the input neurons along the AP axis in different areas of the ipsilateral PAL.

In the PAL, the input neurons were found in the ipsilateral substantia innominata (SI), magnocellular nucleus (MA) and medial septal complex (MSC; Figure 8A). In all of the three brain areas, the distribution patterns of the input neurons were similar between the APC and PPC tracing groups (Figures 8B,C).

## Discussion

The study reported here was undertaken in order to determine the whole-brain direct inputs to two main types of neurons in different PC subareas. Our results are consistent with many previous tracing studies using traditional tracers, but we revealed cell-type-specific inputs to the APC and PPC and quantitatively compared the input proportions. Our results showed that both types of neurons in the APC and PPC integrate extensive inputs from numerous brain areas across the whole brain. In addition, the input patterns are similar for different PC cell types, but they are diverse for different PC subareas. The most prominent differences between the different PC subareas are that the APC received a higher proportion of inputs from the OLF, but lower proportions of inputs from the HPF and CNU than the PPC did.

### Cell-Type-Specific Inputs to the PC

The PC comprises glutamate releasing principal neurons and GABA-releasing interneurons. Previous electrophysiology studies demonstrated that, both principal neurons and interneurons in the PC may show consistent excitatory or inhibitory responses to receptor-specific pharmacologic stimuli or pathway-specific photo genetic stimuli (Tseng and Haberly, 1989; Luna and Morozov, 2012; Sadrian and Wilson, 2015). For instance, activating the PPC projecting basolateral amygdalar nucleus (BLA) neurons can induce excitatory postsynaptic currents (EPSC) on both principal neurons and interneurons of the PPC (Luna and Morozov, 2012), suggesting that both the principal neurons and interneurons of the PC may receive excitatory inputs from the BLA. Our results showed that, in both the APC and PPC, the excitatory Vglut2+ neurons and inhibitory Gad2+ neurons share almost similar input sources, signifying that direct inputs to the PC may target both the excitatory and inhibitory neurons. The diversity of cellular targets within the PC contributes to complex effects on information encoding. For instance, it has been reported that activating the MOB or LOT induces rapid excitation and short time delay feedforward inhibition on the PC principal neurons, with the feedforward inhibition shaping the stimulus receptive fields of the PC (Stokes and Isaacson, 2010; Suzuki and Bekkers, 2012; Large et al., 2016). However, there is still no clear consensus on how these two types of neurons in the PC are connected by their concurrent inputs. In addition, we also found that the excitatory Vglut2+ neurons and inhibitory Gad2+ neurons in the PC share approximately similar proportions of whole-brain inputs from most input sources of them. This is similar to many tracing results from other brain areas that different types of neurons within a certain brain area share similar input patterns across the whole brain (Zhang et al., 2017; Ährlund-Richter et al., 2019; Cai et al., 2019). While it should be noted that, different types of PC neurons may be distinct in their cell morphology, layer distributions, neural circuits and neural response characteristics (Suzuki and Bekkers, 2006, 2011; Diodato et al., 2016; Large et al., 2016). In our studies, we were just concern with the input connectivity of two types of PC neurons, the excitatory Vglut2+ neurons and inhibitory Gad2+ neurons, however, it still needs to be determined if all types of PC neurons share similar input patterns, although different PC subareas show distinct features in their input patterns.

### Input Patterns to Distinct Subareas of the PC

The PC is one key cortical region in the brain responsible for olfactory information processing. Our results revealed that, for both types of neurons, the APC and PPC received dominant inputs from the OLF. While obviously, the APC received high proportions of inputs from the MOB, AON and AOB than the PPC did. Our results are consistent with previous tracing studies using traditional tracers, for instance, mitral/tufted cells in the MOB send denser axons to the APC than to the PPC (Igarashi et al., 2012), and the APC is innervated heavily by the AON (Kowiański et al., 1999). Similar conclusions were also drawn in some electrophysiology studies, for instance, it has been established that the percentages of odor nonresponsive PC neurons were increased from anterior to posterior (Litaudon et al., 2003). The MOB and AON are key nodes in the bottom-up olfactory information transfer process (Shipley and Ennis, 1996), so does the AOB. The heavy peripheral olfactory innervation to the APC suggests that the APC may be more sensitive to peripheral odor stimuli and inclined to integrate olfactory gestalts to generate odor perception (Morrow et al., 2000). In addition, we also noted that over half of the ipsilateral OLF inputs to the PPC came from the ipsilateral APC. A previous study demonstrated that by using the GABA(B) receptor agonist to attenuate PC associational inputs, pattern separation of within-category odors is interfered within the PPC (Bao et al., 2016), meaning that the neural activities in the PC, especially the PPC, may strongly be affected by their associational connections. It could be speculated that the PPC may be in higher associative functions. Besides, it is noteworthy that, although the PC is traditionally defined as a part of the main olfactory pathway, our results showed that the PC received a considerable amount of inputs from the AOB and COApm, which are two major parts of the accessory olfactory system. It has been revealed by previous studies that the AOB sends sparse axons to the APC (Kang et al., 2011; Gutiérrez-Castellanos et al., 2014), thus the APC could respond to some pheromone odorants (Pfaus et al., 2009; Schneider et al., 2016). We extend on the findings of previous studies that, the APC received more AOB inputs than the PPC did, while the PPC received more COApm inputs than the APC did. Our findings provide an anatomical basis that may help elucidate the different roles of APC and PPC in processing vomeronasal information. The main and accessory olfactory systems are believed to function complementarily when they respond to some chemical stimuli. The convergence of olfactory and vomeronasal information in the PC may therefore, help to compose a complete map of the chemical environment and play an important role in the mating and survival for animals (Xu et al., 2005; Martínez-Ricós et al., 2008; Martínez-García et al., 2009).

The PC is not only an information integrator of peripheral olfactory inputs but also a central node in a larger cognitive network involving cortical and limbic connections. Consistent with previous axon tracing studies (Majak et al., 2004; Illig, 2005), our results showed that the isocortex and HPF (a key part of the limbic system) inputs innervated differently on the two PC subareas. The APC received heavy inputs from several brain areas within the isocortex, while the PPC received heavy inputs from the HPF. One of the main isocortex inputs to the APC arise from the ORB, a high order associative cortex integrating multimodal sensory information (Gottfried and Dolan, 2003), which involves in learning and representing information about behavior significance and the associated contextual cue (Bowman et al., 2012; Howard and Gottfried, 2014). The innervation from the ORB to the APC has been reported to play a role in promoting information encoding about odor values or nonolfactory contextual cues in olfactory associated behaviors, and modulating odor response properties of the APC neurons (Schoenbaum and Eichenbaum, 1995; Zinyuk et al., 2001; Roesch et al., 2007; Strauch and Manahan-Vaughan, 2018). Besides the direct cortical connections, the PC also connects with cortical areas indirectly through the TH, especially through the mediodorsal thalamic nucleus (MTN). The MTN, a brain area that is believed to modulate and coordinate activities in the primary sensory system and high order cortical areas (Mease et al., 2016; Courtiol et al., 2019), innervated more heavily to the APC than to the PPC. It could be speculated that the heavy cortical and thalamocortical innervation to the APC may help in forming and recalling associations between odor stimuli, contextual cues, and behavioral outcomes, the multisensory information converging in the APC may facilitate the preprocessing and generating of expectations of incoming olfactory information. In contrast, the limbic system, including the LEC, ventral HIP and AMY, innervate more heavily to the PPC than to the APC (Johnson et al., 2000; Majak et al., 2004). The limbic system has been implicated in a variety of emotional, cognitive and memory processes. For instance, the LEC involves in olfactory discrimination learning and olfactory related associative multimodal memory integration (Chapuis et al., 2013); while the AMY is thought to encode innate and learned odor values and odor intensity, especially that associated to fear and anxiety (Anderson et al., 2003; Sadrian and Wilson, 2015). Both the LEC and AMY have been proved to modulate odor coding in the PC (Anderson et al., 2003; Mouly and Di Scala, 2006; Chapuis et al., 2013; Sadrian and Wilson, 2015). Besides, although the innervation from the ventral HIP to the PC has rarely been studied, perhaps this is due to the low infection efficiency of the traditional tracers and the difficulty to distinguish the axon terminal with pass-by fibers in axons tracing studies. The ventral HIP has been found to innervate strongly to the AON and modulate olfactory sensitivity (Aqrabawi et al., 2016). In addition, the LEC, ventral HIP and AMY are all known to be susceptible to seizures (Mohapel et al., 1996; Vismer et al., 2015; Bui et al., 2018), and all of them connect closely with the PPC, implying that the PPC may be one of the key nodes for seizure spreading (Vismer et al., 2015). Combining the findings of previous studies and our tracing results, it could be speculated that the heavy innervation from the limbic system to the PPC may provide a route by which the animal’s emotional states guide the information processing and memory formation in the PPC.

In addition, the PC also receives a variety of neuromodulatory innervation. Consistent with previous tracing studies using traditional tracers (Haberly and Price, 1978; Kowiański et al., 1999), our tracing studies showed that both the APC and PPC were innervated heavily by the PAL (a brain area belongs to the BF). Together with a previous immunochemistry study which reported that most of the PC-projecting neurons in the BF are choline acetyltransferase positive (Woolf et al., 1984), we concluded that the APC and PPC receive heavy cholinergic inputs from the PAL. The cholinergic inputs to the PC have been suggested play a role in modulating neural excitability and synaptic plasticity of the PC in a state-dependent manner (Barkai and Hasselmo, 1997; Chapuis and Wilson, 2013), high arousal or attention enhances acetylcholine release (Hasselmo and McGaughy, 2004), while disruption of cholinergic activity in the PC impairs odor discrimination and associative memory (Wirth et al., 2000; Fletcher and Wilson, 2002). Except for the PAL inputs, we also found sparsely labeled neurons located in the LC, VTA, and DR. These brain areas are suggested to support noradrenergic, dopaminergic and serotonergic innervation respectively, and play a nonnegligible function in shaping information processing and synaptic plasticity in the PC (Bouret and Sara, 2002; Fletcher and Chen, 2010; Narla et al., 2015). Consistent with the previous axon tracing studies using traditional tracers (De Olmos and Heimer, 1980; Datiche et al., 1995), we found that the APC received obviously more DR inputs than the PPC did (data not shown). Although the role that the serotonergic system plays in olfactory processing within the PC is not well known, it is possible that the serotonergic neuromodulation may be implicated in enhancing the signal-to-noise ratio of odor inputs in the APC (Fletcher and Chen, 2010), because a previous electrophysiology study reported that activation of DR serotonin neurons may inhibit spontaneous activities in the APC, but not influence the odor induced response (Lottem et al., 2016).

### Contralateral Inputs to the PC

Olfactory information integration between the bilateral hemispheres of the brain is crucial for animals to precisely discriminate or localize the odors (Kucharski and Hall, 1988; Rajan et al., 2006; Yan et al., 2008; Esquivelzeta Rabell et al., 2017). The PC is a bilateral structure with a strong reciprocal interconnection *via* the anterior commissure (Martin-Lopez et al., 2018). A previous electrophysiology study showed that the APC responds to odors presented to either the ipsilateral or contralateral nostril (Wilson, 1997). In our study, we found that the commissural inputs of both the APC and PPC mainly arose from the contralateral OLF, implying that the PC may integrate olfactory information from bilateral hemispheres of the brain. In accordance with previous axons tracing studies (Haberly and Price, 1978), we found that, compared with the PPC, the APC received more commissural inputs, especially from the contralateral AON, a brain area which is believed to generate olfactory gestalts (Shipley and Ennis, 1996; Brunjes et al., 2005), suggesting a role of the APC in odor identity information integration from bilateral hemispheres. Besides the contra-AON inputs, we also noted that both the APC and PPC received commissural inputs from the contralateral APC, especially from the contralateral rAPC. The APC not only encodes odor perception, but also encodes odor associated values or context (Roesch et al., 2007; Wilson and Sullivan, 2011). The commissural connections between the bilateral APC, may suggest that not only the odor identity information, but also the odor associated value or context information may be exchanged between the bilateral hemispheres. Furthermore, the rAPC is considered as a seizure susceptible area (Piredda and Gale, 1985), the close connections between the bilateral PC may play a role in seizure spreading. In fact, many previous behavioral studies have shown that olfactory information could be shared between the two hemispheres in some innate odor-driven behaviors such as odor habituation, and simple behavior tasks, such as odor associated preference and coarse odor discrimination task (Kucharski and Hall, 1987, 1988; Mainland et al., 2002; Yan et al., 2008), but not in fine odor discrimination task (Feng and Zhou, 2019). This could be due to the odor identification relying more on the highly commissural APC network, while the fine odor discrimination may be depending more on the highly associative but less commissural PPC network.

In summary, the whole-brain direct inputs to excitatory and inhibitory neurons in different PC subareas were mapped in this study. Although the input patterns are similar for different cell types, they are diverse for different PC subareas. The findings revealed that the PC integrates extensive inputs from numerous brain areas across the whole brain, and the APC and PPC are innervated differently by the OLF and HPF, which may provide new insights for further study into the diverse functions of the PC.

## Data Availability Statement

The raw data supporting the conclusions of this article will be made available by the authors, without undue reservation, to any qualified researcher.

## Ethics Statement

The animal study was reviewed and approved by The Animal Care and Use Committees at the Wuhan Institute of Physics and Mathematics, Chinese Academy of Sciences.

## Author Contributions

LW, ZZ, and FX designed the experiments. LW and JC performed experiments. LW analyzed the data. RH was involved in conceptual formation. LW, ZZ, AM, QL, and FX contributed to manuscript writing. LW generated the figures.

## Conflict of Interest

The authors declare that the research was conducted in the absence of any commercial or financial relationships that could be construed as a potential conflict of interest.
