# Supplementary material for: Cell-Type-Specific Whole-Brain Direct Inputs to the Anterior and Posterior Piriform Cortex
Source: Front Neural Circuits. 2020 Feb 7;14:4. doi: 10.3389/fncir.2020.00004 (PMC7019026; doi:10.3389/fncir.2020.00004)
Supplement: Supplementary file 1 [file Data_Sheet_1.PDF]

Supplementary Material

Supplementary Table 1. Abbreviations

Definition	Abbreviation
**Isocortex**	
Orbital area	ORB
Agranular insular area	AI
Somatomotor areas	MO
Perirhinal area	PERI
Somatosensory areas	SS
**Olfactory region**	**OLF**
Main olfactory bulb	MOB
Accessory olfactory bulb	AOB
Anterior olfactory nucleus	AON
Dorsal part of the AON	AONd
Lateral part of the AON	AONl
Medial part of the AON	AONm
Posteroventral part of the AON	AONpv
Piriform cortex	PC
Anterior piriform cortex	APC
Rostral part of the APC	rAPC
Caudal part of the APC	cAPC
Posterior piriform cortex	PPC
Taenia tecta	TT
Dorsal part of the TT	TTd
Ventral part of the TT	TTv
Nucleus of the lateral olfactory tract	NLOT
Cortical amygdalar area	COA
Anterior part of the COA	COAa
Posterolateral part of the COA	COApl
Posteromedial part of the COA	COApm
Postpiriform transition area	TR
Piriform-amygdalar area	PAA
**Hippocampal formation**	**HPF**
Hippocampal region	HIP
Retrohippocampal region	RHP

Lateral entorhinal cortex	LEC
**Cortical subplate**	**CTXsp**
Endopiriform nucleus	EP
Basomedial amygdalar nucleus	BMA
Basolateral amygdalar nucleus	BLA
**Cerebral nuclei**	**CNU**
Striatum	STR
Anterior amygdalar area	AAA
Central amygdalar area	CEA
Medial amygdalar area	MEA
Pallidum	PAL
Substantia innominate	SI
Magnocellular nucleus	MA
Medial septal complex	MSC
**Interbrain**	**IB**
Thalamus	TH
Midline group of the dorsal thalamus	MTN
Hypothalamus	HY
**Midbrain**	**MB**
Ventral tegmental area	VTA
Dorsal raphe nucleus	DR
**Hindbrain**	**HB**
Locus coeruleus	LC
**Others**	
Lateral olfactory tract	LOT
Basal forebrain	BF
Amygdala	AMY
Anterior to posterior	AP
Adeno-associated virus	AAV
Rabbit virus	RV
γ-aminobutyric acid	GABA
Excitatory postsynaptic currents	EPSC
Phosphate buffered saline	PBS
Paraformaldehyde	PFA

Supplementary Table 2. Statistical analyses

Figure	Target	Data values	P values	Methods
2B	OLF	77.44%±0.96% for $APC^{Vglut2+}$ vs $PPC^{Vglut2+}$, 68.74%±1.43%	<0.001	T-tests
	OLF	82.93%±1.54% for APC^{Gad2+} vs PPC^{Gad2+}, 57.07%±4.17%	<0.001	T-tests
	HPF	2.00%±0.43% for $APC^{Vglut2+}$ vs $PPC^{Vglut2+}$, 8.37%±1.38%	0.001	T-tests
	HPF	0.89%±0.29% for APC^{Gad2+} vs PPC^{Gad2+}, 7.24%±1.74%	0.002	T-tests
	CNU	4.07%±0.34% for $APC^{Vglut2+}$ vs $PPC^{Vglut2+}$, 8.44%±0.50%	<0.001	T-tests
	CNU	5.80%±0.85% for APC^{Gad2+} vs PPC^{Gad2+}, 15.81%±1.79%	0.002	T-tests
2C	Ipsi-MOB	15.43%±1.38% for $APC^{Vglut2+}$ vs $PPC^{Vglut2+}$, 11.63%±0.96%	0.048	T-tests
	Ipsi-MOB	37.50%±5.39% for APC^{Gad2+} vs PPC^{Gad2+}, 5.67%±2.53%	<0.001	T-tests
	Ipsi-PC	20.79%±1.77% for $APC^{Vglut2+}$ vs $PPC^{Vglut2+}$, 44.26%±1.73%	<0.001	T-tests
	Ipsi-PC	22.71%±6.82% for APC^{Gad2+} vs PPC^{Gad2+}, 42.55%±4.50%	0.044	T-tests
	Ipsi-AON	30.77%±1.62% for $APC^{Vglut2+}$ vs $PPC^{Vglut2+}$, 6.96%±1.02%	<0.001	T-tests
	Ipsi-AON	14.33%±6.88% for APC^{Gad2+} vs PPC^{Gad2+}, 1.83%±0.41%	0.083	T-tests
	Ipsi-EP	7.85%±0.74% for $APC^{Vglut2+}$ vs $PPC^{Vglut2+}$, 7.81%±0.27%	0.956	T-tests
	Ipsi-EP	8.57%±1.03% for APC^{Gad2+} vs PPC^{Gad2+}, 12.36%±2.29%	0.115	T-tests
	Ipsi-RHP	1.73%±0.38% for $APC^{Vglut2+}$ vs $PPC^{Vglut2+}$, 5.87%±0.98%	0.003	T-tests
	Ipsi-RHP	0.89%±0.29% for APC^{Gad2+} vs PPC^{Gad2+}, 6.22%±1.41%	0.002	T-tests
	Contra-AON	13.06%±1.39% for $APC^{Vglut2+}$ vs $PPC^{Vglut2+}$, 0.25%±0.11%	<0.001	T-tests
	Contra-AON	4.09%±2.50% for APC^{Gad2+} vs PPC^{Gad2+}, 0.13%±0.08%	0.164	T-tests
4B	MOB	24.68%±1.37% for $APC^{Vglut2+}$ vs $PPC^{Vglut2+}$, 17.20%±1.01%	0.001	T-tests
	AON	28.55%±2.25% for $APC^{Vglut2+}$ vs $PPC^{Vglut2+}$, 10.12%±1.33%	<0.001	T-tests
	PC	30.45%±2.65% for $APC^{Vglut2+}$ vs $PPC^{Vglut2+}$, 64.97%± 1.34%	<0.001	T-tests
	TT	8.45%±1.62% for $APC^{Vglut2+}$ vs $PPC^{Vglut2+}$, 3.10%±0.32%	0.009	T-tests
	NLOT	3.26%±0.82% for $APC^{Vglut2+}$ vs $PPC^{Vglut2+}$, 0.69%±0.21%	0.013	T-tests
	AOB	2.41%±0.64% for $APC^{Vglut2+}$ vs $PPC^{Vglut2+}$, 0.28%±0.16%	0.009	T-tests
	COA	1.14%±0.14% for $APC^{Vglut2+}$ vs $PPC^{Vglut2+}$, 2.35%±0.30%	0.004	T-tests
4E	COAa	76.62%±8.13% for $APC^{Vglut2+}$ vs $PPC^{Vglut2+}$, 46.68%±10.67%	0.050	T-tests
	COApl	18.32%±6.99% for $APC^{Vglut2+}$ vs $PPC^{Vglut2+}$, 24.70%±8.48%	0.575	T-tests
	COApm	5.06%±2.39% for $APC^{Vglut2+}$ vs $PPC^{Vglut2+}$, 28.63%±7.73%	0.016	T-tests
5B	AON	86.63%±1.66% for $APC^{Vglut2+}$ vs $PPC^{Vglut2+}$, 9.09%±3.94%	<0.001	T-tests
	PC	30.45%±2.65% for $APC^{Vglut2+}$ vs $PPC^{Vglut2+}$, 64.97%±1.34%	<0.001	T-tests
	NLOT	3.26%±0.82% for $APC^{Vglut2+}$ vs $PPC^{Vglut2+}$, 0.69%±0.21%	0.013	T-tests
5C	AON	0.7898±0.1277for $APC^{Vglut2+}$ vs $PPC^{Vglut2+}$, 0.0315±0.0126	0.001	T-tests
	PC	0.1066±0.0226for $APC^{Vglut2+}$ vs $PPC^{Vglut2+}$, 0.0344±0.0067	0.015	T-tests
	NLOT	0.2153±0.0326 for $APC^{Vglut2+}$ vs $PPC^{Vglut2+}$, 2.7267±0.7597	0.007	T-tests

	$APC^{Vglut2+}$	rAPC vs cAPC vs PPC	<0.001	ANOVA
	$PPC^{Vglut2+}$	rAPC vs cAPC vs PPC	<0.001	ANOVA
5E	$APC^{Vglut2+}$	87.74%±2.44% for rAPC vs cAPC, 11.50%±2.23%	<0.001	B-tests
	$APC^{Vglut2+}$	11.50%±2.23% for cAPC vs PPC, 0.76%±0.41%	0.004	B-tests
	$PPC^{Vglut2+}$	54.59%±7.09% for rAPC vs cAPC, 36.48%±4.37%	0.082	B-tests
	$PPC^{Vglut2+}$	36.48%±4.37% for cAPC vs PPC, 8.92%±3.57%	0.006	B-tests
6B	ORB	37.88%±1.84% for $APC^{Vglut2+}$ vs $PPC^{Vglut2+}$, 1.34%±0.85%	<0.001	T-tests
	AI	34.47%±3.02% for $APC^{Vglut2+}$ vs $PPC^{Vglut2+}$, 49.19%±4.34%	0.093	T-tests
	MO	11.82%±2.30% for $APC^{Vglut2+}$ vs $PPC^{Vglut2+}$, 1.75%±0.43%	0.002	T-tests
	PERI	5.02%±1.05% for $APC^{Vglut2+}$ vs $PPC^{Vglut2+}$, 15.58%±3.69%	0.021	T-tests
	SS	2.14%±0.82% for $APC^{Vglut2+}$ vs $PPC^{Vglut2+}$, 15.21%± 4.22%	0.012	T-tests
7B	HIP	11.81%±4.86% for $APC^{Vglut2+}$ vs $PPC^{Vglut2+}$, 30.04%±2.03%	0.006	T-tests
	RHP	88.19%±4.86% for $APC^{Vglut2+}$ vs $PPC^{Vglut2+}$, 69.96%±2.03%	0.006	T-tests
8B	SI	47.98%±2.35% for $APC^{Vglut2+}$ vs $PPC^{Vglut2+}$, 43.16%±2.37%	0.178	T-tests
	MA	29.27%±2.70% for $APC^{Vglut2+}$ vs $PPC^{Vglut2+}$, 30.91%±4.05%	0.473	T-tests
	MSC	22.64%±2.04% for $APC^{Vglut2+}$ vs $PPC^{Vglut2+}$, 23.87%±2.88%	0.733	T-tests

T-tests: Student’s t-tests; ANOVA: one-way ANOVA tests; B-tests: Bonferroni tests

Supplementary Figure 1

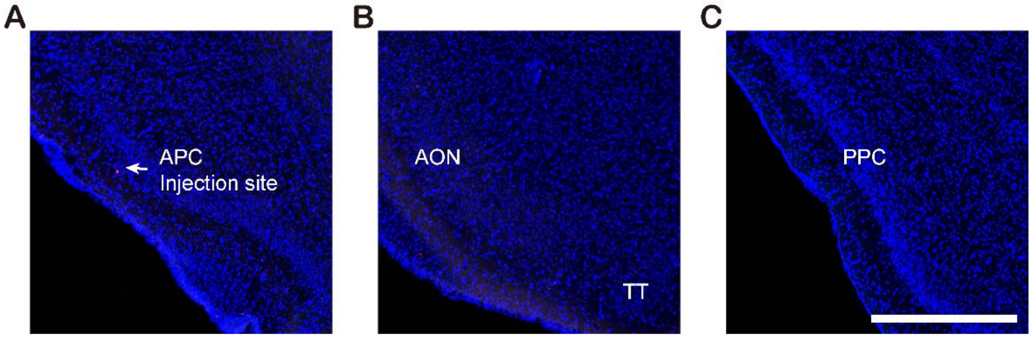

Supplementary Figure 1. Representative images of coronal brain sections after injecting AAV-Dio-GFP-TVA and AAV-Dio-RVG and RV-EnvA-ΔG-dsRed into wild-type mice. (A) Coronal brain section containing the injection site (APC) showed a very limited number of EnvA-dsRed positive neurons. (B, C) Coronal brain sections containing the AON, TT and PPC showed no RV labeled neurons. Scale bar: 500 µm.
